# Aconitase: To Be or not to Be Inside Plant Glyoxysomes, That Is the Question

**DOI:** 10.3390/biology9070162

**Published:** 2020-07-12

**Authors:** Luigi De Bellis, Andrea Luvisi, Amedeo Alpi

**Affiliations:** 1 Department of Biological and Environmental Sciences and Technologies, University of Salento, Via Prov. le Monteroni, I-73100 Lecce, Italy; andrea.luvisi@unisalento.it; 2 Approaching Research Educational Activities (A.R.E.A.) Foundation, I-56126 Pisa, Italy; amedeo.alpi@gmail.com

**Keywords:** aconitase, malate dehydrogenase, glyoxylate cycle, glyoxysomes, peroxisomes, β-oxidation, gluconeogenesis

## Abstract

After the discovery in 1967 of plant glyoxysomes, aconitase, one the five enzymes involved in the glyoxylate cycle, was thought to be present in the organelles, and although this was found not to be the case around 25 years ago, it is still suggested in some textbooks and recent scientific articles. Genetic research (including the study of mutants and transcriptomic analysis) is becoming increasingly important in plant biology, so metabolic pathways must be presented correctly to avoid misinterpretation and the dissemination of bad science. The focus of our study is therefore aconitase, from its first localization inside the glyoxysomes to its relocation. We also examine data concerning the role of the enzyme malate dehydrogenase in the glyoxylate cycle and data of the expression of aconitase genes in Arabidopsis and other selected higher plants. We then propose a new model concerning the interaction between glyoxysomes, mitochondria and cytosol in cotyledons or endosperm during the germination of oil-rich seeds.

## 1. Introduction

**Figure 1:**
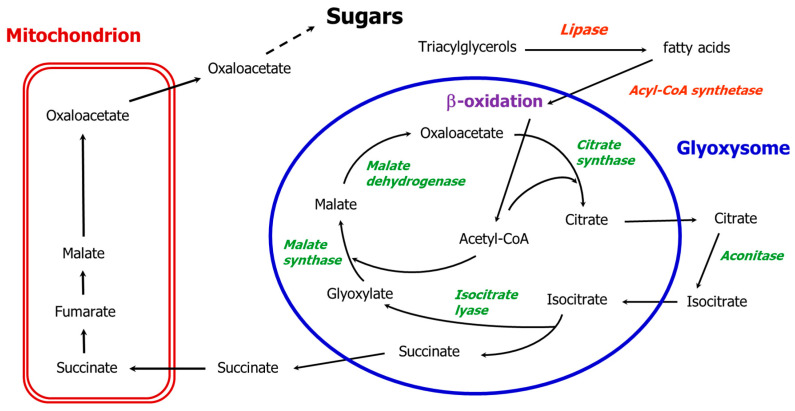
The path of carbon in the classical glyoxylate cycle except for aconitase in the cytosol. Black arrows indicate glyoxylate cycle reactions; the five enzymes of the glyoxylate cycle are in green.

Glyoxysomes are specialized types of plant peroxisomes containing glyoxylate cycle enzymes, which participate in the conversion of lipids to sugar during the early stages of germination in oilseeds. Glyoxysomes were originally named by Harry Beevers and colleagues [1,2] following Christian De Duve’s definition of peroxisomes [3]. Higher plant glyoxysomes are sites of the β-oxidation of fatty acids and determine the production of succinate by means of glyoxylate cycle enzymes. Succinate diffuses out to be converted into oxaloacetate in the mitochondria, and oxaloacetate is then used in the cytoplasm for the synthesis of glucose by gluconeogenesis (the classical glyoxylate cycle, Figure 1). The conversion of fats into carbohydrates while avoiding the decarboxylative steps of the Krebs cycle is unique to glyoxysomes [1,4].

Thus, the glyoxylate cycle is fundamental to the germination of seeds rich in lipid reserves, and for the mobilization of lipids during tissue development, senescence, starvation and tissue culture [5].

All of the five key enzymes of the glyoxylate cycle, aconitase, isocitrate lyase, malate synthase, malate dehydrogenase and citrate synthase, were originally assumed to be located in glyoxysomes [4], but aconitase has always been viewed as an exception, due to either rapid solubilization or inactivation during the sucrose gradient separation [4]. In the 1990s, two research groups focused on aconitase and independently demonstrated its absence in glyoxysomes [6,7,8,9]. However, some research has continued to consider aconitase not only as an enzyme involved in the mitochondrial Krebs cycle but also as a glyoxylate cycle enzyme localized in glyoxysomes, e.g., [10,11,12,13,14]. However, in other research aconitase is not cited as part of the glyoxylate cycle [15], and in 2020 it was defined as a “presumably non-peroxisomal enzyme” in the high-ranking journal New Phytologist [16]. Thus, the research into aconitase should be reviewed to further clarify the issue and to inform researchers who, in the digital age, may rarely consult scientific articles published more than twenty years ago.

## 2. The Glyoxysome Story

Glyoxysomes were first isolated from the endosperm of young castor bean seedlings and were named by Beevers’ group of researchers in 1967/1968 [1,2]. They represent a class of highly specialized microbodies (peroxisomes) and are characterized by an equilibrium density of approximately 1.25 g cm^3^ after sucrose gradient separation. They contain marker enzymes such as catalase, urate oxidase, glycolate oxidase and the enzymes of the glyoxylate cycle and of the β-oxidation of fatty acids [17]. 

Glyoxysomes are typically present in the cotyledons or endosperm of germinating fatty seeds, where the β-oxidation system for fatty acid degradation produces acetyl CoA, which by entering the glyoxylate cycle and avoiding the Krebs cycle direct the carbon flow toward sugar synthesis. In the absence of photosynthesis, the glyoxylate cycle enables the conversion of water insoluble reserves such as lipids into soluble sugars that can be mobilized towards other actively growing tissues. The specific role of the glyoxylate cycle is to condense two molecules of acetyl CoA into a four-carbon molecule (succinate), thus avoiding the respiration of acetyl CoA within the reserve tissue. This cycle was originally suggested to be localized within glyoxysomes by Breidenbach et al. [2], who drew it with a dashed line (due to the lack of data confirming the association of aconitase with glyoxysomes) for the isomerization of citrate to isocitrate. They hypothesized a re-oxidation of the NADH produced by the glyoxysomal malate dehydrogenase via the reduction of glyoxylate to glycolate by the enzyme glyoxylate reductase. We refer to this cycle, now outdated, as the “classical glyoxylate cycle” (Figure 1).

Various scientific articles on the glyoxylate cycle were published in the following years, but Cooper and Beevers [4] failed to demonstrate the localization of aconitase within glyoxysomes. After the sucrose gradient fractionation, they found that under 10% of the total aconitase activity was recovered along the gradient in the glyoxysomal fractions. To explain the loss of activity following the organelles’ separation procedure, the authors proposed rapid solubilization of the enzyme and its inactivation during the relatively long separation procedure [4]. This explanation was then confirmed in other studies, which suggested the low aconitase activity in the glyoxysomal fractions was an “extensive solubilization” [17], which was then supported in research reviews [18,19,20,21,22,23]. Thus, in the following years, aconitase was considered to be present within glyoxysomes, but was impossible to detect in purified organelle fractions due to extreme solubilization or inactivation. Hence, although the glyoxylate cycle has been identified in plant organs other than seedlings such as senescent leaves, flowers and fruits [24,25,26,27,28,29], the presence of aconitase in organelles has not been investigated, because the presence of the two marker enzymes isocitrate lyase and malate synthase was considered sufficient for a functional glyoxylate cycle. 

Roland Douce’s group then focused its attention on plant aconitase, first characterizing a mitochondrial aconitase [30], and then demonstrating in 1987, around twenty years after the discovery of the glyoxylate cycle, that aconitase activity in sycamore cells is associated with both the mitochondrial and cytosolic fraction [31], thus laying the foundation for a new interpretation of aconitase localization. However, cytosolic and mitochondrial aconitase eluted almost at an identical position in DEAE-trisacryl column chromatography. Douce’s group continued their research into aconitase and showed that an enzyme purified by potato tuber mitochondria was rapidly inactivated in the presence of hydrogen peroxide [32]. This led to new concerns about the expected presence of aconitase within glyoxysomes, as these are sites where H_2_O_2_ is constantly produced during the β-oxidation process. In 1991, Verniquet et al. [32] concluded that aconitase was not present in glyoxysomes, and that a step in the cytosol in which the activity of the enzyme aconitase is particularly high is necessary for the functionality of the glyoxylate cycle [31].

The work of Verniquet et al. [32] encouraged others to further examine aconitase, as the only enzyme of the glyoxylate cycle that has not been purified or extensively studied [17,18,19,20,21,22,23]. De Bellis et al. [33] purified and characterized aconitase isoforms from pumpkin cotyledons without detecting aconitase activity in glyoxysomes after sucrose gradient fractionation. Three aconitase isoforms were detected and two (ACO I and ACO II) were purified, demonstrating that the enzyme consists of a monomer of approximately 98 kDa. The two aconitase isoforms were characterized by a pI between 4.8 and 5, but their subcellular localization was not clearly determined [33]. 

Douce’s group independently published a paper around the same time, with the aim of establishing whether aconitase was present in the peroxisomes (glyoxysomes) of plant cells [6]. Mitochondria and peroxisomes were purified from castor bean endosperm using a step sucrose gradient and from the potato tuber using a self-generating Percoll gradient. Both the isolated castor bean glyoxysomes and purified potato tuber peroxisomes appeared devoid of aconitase activity [6]. Mitochondrial aconitase was then purified from the potato tuber to obtain antibodies. A protein of approximately 94 kDa was observed in the mitochondrial fractions and in a supernatant practically free of mitochondrial marker activities, but not in the castor bean endosperm glyoxysomes and potato tuber peroxisomes. These results clearly suggest that the presence of aconitase is restricted to mitochondria and cytosol, and it is an enzyme essentially absent in glyoxysomes or peroxisomes [6]. The authors regarded this as “surprising, because it is generally believed that the glyoxysomal aconitase is part of the glyoxylate cycle”. Therefore, in order for the glyoxylate cycle to function, the citrate must exit the glyoxysomes and re-enter as isocitrate after conversion by the cytosolic aconitase [6]. Thus, exactly 25 years after the first hypothesis concerning aconitase localization [2], a new and clearer view emerged.

An Italian–Japanese group developed an approach similar to that of Courtois-Verniquet et al. [6], by obtaining specific antibodies against pumpkin aconitase through an affinity purification of the antiserum with purified aconitase [7]. Antibodies were used to demonstrate that the presence of aconitase has a pattern consistent with the enzymatic activity of both aconitase and isocitrate lyase (as the main glyoxysomal marker) during the development of pumpkin seedlings first in darkness and then in light (thus inducing autotrophic growth), which supported the hypothesis of aconitase participation in the glyoxylate cycle, while the absence of aconitase in purified fractions of glyoxysomes were confirmed through immunoblots [7]. Thus, additional reliable evidence that aconitase is not present within the glyoxysomes was provided by a second independent research group. 

The Italian–Japanese collaboration continued their research into aconitase and published additional articles [8,9,34]. They first confirmed the presence of three aconitase isoforms in pumpkin seedlings [33] and that the isoform ACO I appeared to be involved in the glyoxylate cycle, as it accounts for most of the aconitase activity in the cotyledons of pumpkin seedlings grown in the dark [8]. In addition, aconitase was barely detectable in roots, hypocotyls and green leaves, while ACO II was revealed to be the mitochondrial isoform, and ACO I and ACO III were cytosolic isoforms [8]. The results reported between 1993 and 1995 [8,33] indicate that the pI of the pumpkin cytosolic aconitase part of the glyoxylate cycle (ACO I) is 5.0, and that of the mitochondrial aconitase 4.8, and thus appear to be slightly lower than the pI of ACO III. Hayashi et al. [9] achieved a cDNA of 3.145 bp after a screening of a cDNA library obtained from etiolated pumpkin cotyledons, which the open reading frame encodes for a polypeptide of 97.893 Da (in agreement with the previously indicated apparent molecular mass of 98 kDa [33]), which appeared to be very similar to iron-responsive element binding proteins (IRE-BPs) cloned from the rabbit, mouse, rat and human [9]. IRE-BPs are proteins that play a key role in the regulation of iron homoeostasis and correspond to animal cytosolic aconitase and bacterial aconitase as they are able to switch to RNA-binding proteins; however, this mechanism does not operate in plants [35].

Studies of glyoxysomes in the following years focused on aspects other than the aconitase enzyme, such as beta oxidation enzymes, targeting and mutant production in Arabidopsis [5,36,37,38,39,40]. In addition, further studies of aconitase demonstrated that tobacco aconitase, like the animal counterpart, is inhibited by nitric oxide (NO), suggesting that NO is also able to modulate the enzyme in plants [41], that the repression of both mitochondrial and cytosolic tomato aconitase has drastic effects on photosynthesis and on fruit yield [42], and that plant aconitase acts as a mediator of oxidative stress and the regulation of cell death [43], although the role of the IRE binding protein has not been confirmed [35].

Mettler and Beevers reviewed the glyoxylate cycle in 1980 [44] addressing the problem of the re-oxidation of NADH produced by β-oxidation and by the malate dehydrogenase step in the classical glyoxylate cycle, in which one mol of succinate and three mol of NADH are generated (one from the oxidation of malate and two from β-oxidation) for each complete run of the cycle. The glyoxysomes evidently could not re-oxidize NADH, so Mettler and Beevers proposed a complex malate-aspartate shuttle [44]: aspartate and 2-oxoglutarate come out of the mitochondria and enter into glyoxysomes where they are transaminated to form glutamic acid and oxaloacetate. Glutamate is imported into mitochondria while oxaloacetate is in part reduced to malate by malate dehydrogenase with the oxidation of NADH, and in part enters the glyoxylate pathway to generate malate and succinate, which is then imported into the mitochondria. To complete the shuttle, aspartate and 2-oxoglutarate are produced again in the mitochondria via oxidation and transamination reactions. The proposed shuttle involved two different methods of producing malate. First, through a malate dehydrogenase which reduces oxaloacetate to malate, and second, through the classic reaction of the cycle catalyzed by the glyoxysomal marker enzyme malate synthase. Mettler and Beevers [44] thus suggested that within glyoxysomes the enzyme malate dehydrogenase does not catalyze the oxidation of malate to oxaloacetate as originally hypothesized for the classic glyoxylate cycle (Figure 1). 

**Figure 2:**
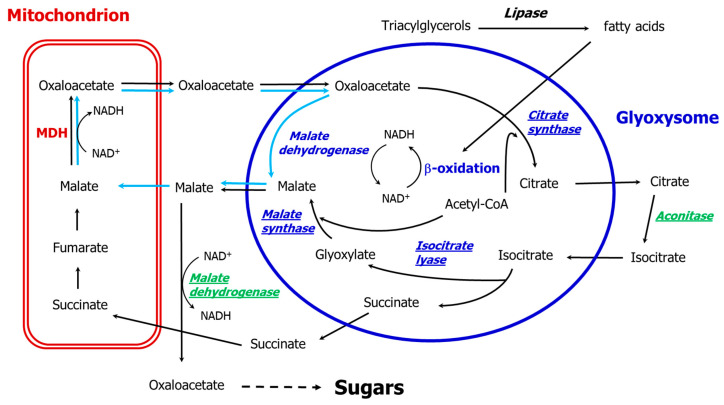
The path of carbon in the glyoxylate cycle after van Roermund et al. [46], Pracharoenwattana et al. [49] and Pracharoenwattana and Smith [5]. Black arrows indicate glyoxylate cycle reactions up to sugars, while light blue arrows indicate the malate/oxaloacetate shuttle transport/reactions; the cytosolic enzymes are given in green, the glyoxysomal enzymes in blue, and the enzymes of the glyoxylate cycle including the cytosolic malate dehydrogenase are underlined. MDH: mitochondrial malate dehydrogenase.

In the early eighties, the peroxisomal membrane was found to be impermeable to NAD and NADH [44,45], thus requiring the re-oxidation of NADH produced via the β-oxidation pathway must take place inside the organelle. The process is analogous to that of the yeast *Saccaromyces cerevisiae*, in which re-oxidation is carried out by peroxisomal malate dehydrogenase (MDH3) and malate is exported to and oxidized in the cytosol by MDH2 [46,47,48]. In both organisms, the peroxisomal malate dehydrogenase is required for β-oxidation and not for the glyoxylate cycle [46,49]. Moreover, the germination and establishment of Arabidopsis are not controlled by β-oxidation as the *mdh1mdh2* double mutant is impaired in β-oxidation but not in the glyoxylate cycle, as it can metabolize ^14^C into sugars [49]. A malate/oxaloacetate shuttle was proposed [5,46,49]: malate comes out of the peroxisome and oxaloacetate re-enters allowing the oxidation of the NADH produced by β-oxidation. This proposition complements and extends the proposal of Mettler and Beevers [44], and means that in terms of the glyoxylate cycle the participating malate dehydrogenase is located outside the glyoxysomes (Figure 2), while inside the organelles a peroxisomal malate dehydrogenase realizes the reduction of oxaloacetate to malate, with the re-oxidation of NADH derived from the β-oxidation pathway. This route enables the oxidation of the NADH produced by the β-oxidation flux, and also explains the role of the glyoxysomal malate dehydrogenase and why this enzyme has always been found within the purified organelles. 

## 3. Aconitase Gene Families in Plants

Thus, the enzyme aconitase appears to be present exclusively in the cytosol and the mitochondria (where part of the Krebs cycle is located). However, in the aconitase polypeptides specific targeting signals have not been identified, neither for peroxisomes [50] nor for mitochondria [51]. Thus, from these data it is not possible to distinguish genes encoding mitochondrial or cytosolic isoforms. In 1995, plant aconitase was first cloned in the cDNA library by Peyrett et al. [52] from *Arabidopsis thaliana* immature pods. They demonstrated that the cloned gene was specifically expressed during germination and pollen and seed maturation, but they could not determine whether a single or multiple genes encode for the mitochondrial and cytosolic aconitases. A few months later, aconitase was cloned from pumpkin by Hayashi et al. [9], who assumed that the cDNA encodes for a cytosolic isoform of aconitase. Many aconitase sequences were then identified, including four putative Arabidopsis aconitases [53], due to the sequencing of numerous plant genomes. Then the functional divergences and evolution of the aconitase family could be investigated [51,54] without resolving the main issue of identifying the genes that encode for mitochondrial or cytosolic aconitase and those involved in the glyoxylate cycle. The products of the three Arabidopsis aconitase genes (*ACO1-ACO3*) detected in Arabidopsis mitochondrial proteomes appeared to be targeted at both cytosol and mitochondria [53,55,56]. Wang et al. [51] recently examined the evolution of aconitase genes from 12 selected land plants (Arabidopsis, maize, soybean, a gymnosperm species, 1 lycophyte and 1 bryophyte), identifying a duplication history of aconitase genes in higher plants, a divergent expression of the genes, and numerous cis-acting elements in the promoter regions. However, they concluded that all land plant aconitases are cytosolic and no mitochondrial isoforms were present in the twelve genomes analyzed. This confirmed that a bioinformatics prediction should be treated with caution, as the conclusions of Wang et al. [51] are not supported by the presence of aconitase activity and aconitase protein in mitochondrial fractions, as several authors previously found in different species both over 20 years ago [4,6,9,17,57] and more recently [58].

In addition, the findings concerning the Arabidopsis mutants of the three aconitase genes have not been conclusive. Arnaud et al. [35] suggested that ACO1 and ACO3 are cytosolic proteins and ACO2 is the mitochondrial aconitase, even though the greatest reduction in mitochondrial aconitase activity was verified for the *aco3* mutant (55%, whereas 20% was registered for *aco1* and *aco2*, in 10-day-old plantlets). The main reduction in total activity was for *aco1*, and an additional indication was that the double mutant *aco1/aco3* caused the abortion of seeds, suggesting the two genes had a fundamental role in embryo formation and seed development [35]. Moeder et al. [43] demonstrated that recombinant Arabidopsis ACO1 binds to the 5ʹ UTR of the chloroplastic CuZn-superoxide dismutase gene (*CSD2*), and that aconitase knockout mutants are more tolerant than the wild type to oxidative stress. They also found that in the *aco1* and *aco2* mutant lines the total aconitase activity was reduced by approximately 20% each, and in *aco3* there was a 70% decrease from that of the wild type, while none of these lines exhibited a delay in germination. These data imply that ACO3 should be the main aconitase in Arabidopsis, and that none of the genes are singularly necessary in the early germination step of Arabidopsis oilseeds. Bernard et al. [59] reported that the activity of cytosolic aconitase was significantly reduced in leaves of Arabidopsis atm (ATP-binding cassette transporters of mitochondria) knockout mutants and conducted a nondenaturing gel electrophoresis of leaf samples from *aco1-aco3* mutants at a pH of 8.6. The ACO1 protein exhibited the lowest level of electrophoretic mobility, followed by ACO3 and ACO2, and an immunoblot analysis of cytosolic and mitochondrial fractions prepared from leaf protoplasts indicated that the ACO1 was cytosolic and ACO2 and ACO3 mitochondrial, which was “in agreement with proteomics data (SUBA—SUB cellular localisation database for Arabidopsis proteins at http://www.plantenergy.uwa.edu.au)”. The SUBA database currently indicates that all three proteins are localized in mitochondria, whereas only one of the prediction tools, the fluorescent protein assay, suggests ACO1 is uniquely localized in the cytosol.

Hooks et al. [60] used several *aco1-aco3* mutant accessions (including only one from Arnaud et al. [35]) and concluded that the cytosolic ACO3 has a major role in citrate metabolism, and that such isoform is part of the classic glyoxylate cycle. During germination, the *ACO3* transcripts peaked at day 1 and the initial growth of *aco3* was reduced in comparison to the wild type and the *aco1* and *aco2* mutants. Hooks et al. [60] thus hypothesized that ACO3 is mainly cytosolic at the first stage of germination, which corresponds to the degradation of the lipid reserves, and is prevalently mitochondrial afterwards. The predictions of the intracellular localization of Arabidopsis aconitase from the public databases are illustrated in Table 1. 

Few studies have focused on cytosolic and mitochondrial aconitase in other plants. Cots and Widmer [57] identified the soybean cytosolic aconitase participating in the glyoxylate cycle in the C1 isoform from the five aconitase isoforms identified in soybean seedlings (two mitochondrial: M1 and M2; three cytosolic: C1, C2 and C3), but six genes are expressed in *Glycine max* [51]. Eprintsev et al. [61] identified one aconitase localized in the cytosol and one in the mitochondria of maize caryopses, with the former less inhibited by H_2_O_2_ and possibly part of the glyoxylate cycle. They later [58] demonstrated the presence of only two active isoforms in corn leaves, one cytosolic and one mitochondrial, with the latter having a higher level of mobility after native electrophoresis, whereas six aconitase genes are present and expressed in *Zea mais* [51].

**Figure 3:**
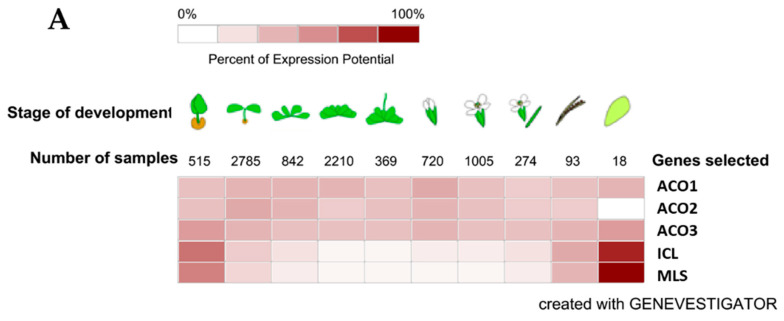

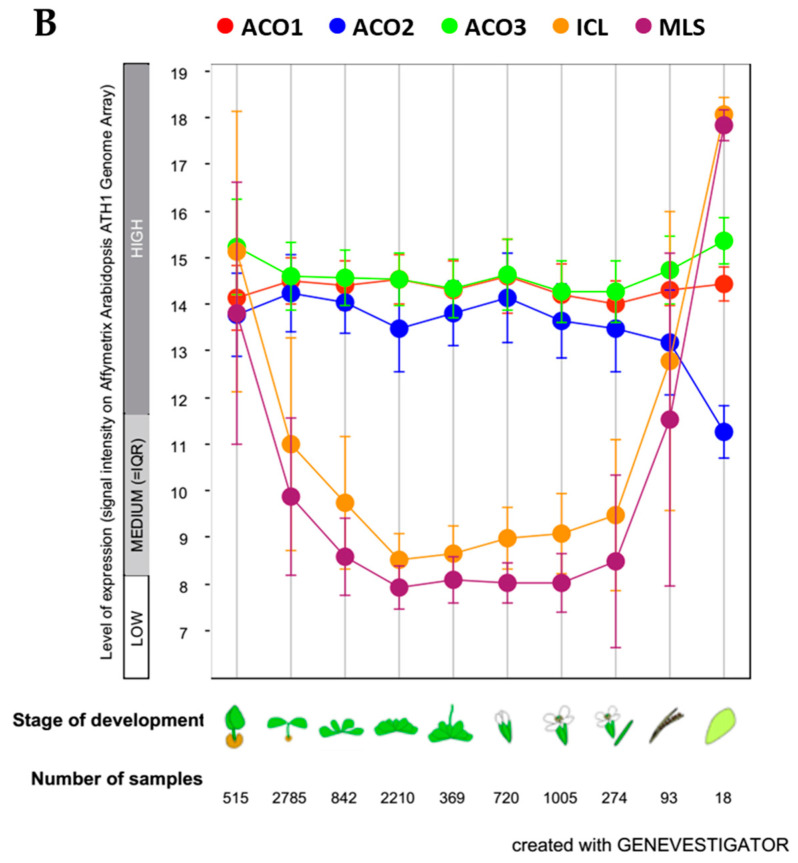
Expression of Arabidopsis genes plotted with Genevestigator (https://genevestigator.com) on Affymetrix Arabidopsis ATH1 Genome Array data. (**A**) Data expressed in a color coding scaled to the expression potential (=the maximum expression a gene reaches across all experiments); ACO1: aconitase 1 (At4g35830); ACO2: aconitase 2 (At4g26970); ACO3: aconitase 3 (At2g05710); ICL: isocitrate lyase (AT3G21720); MLS: malate synthase (At5g03860). (**B**) Data expressed as log2 scale; ●ACO1: aconitase 1 (At4g35830); ●ACO2: aconitase 2 (At4g26970); ●ACO3: aconitase 3 (At2g05710); ● ICL: isocitrate lyase (AT3G21720); ● MLS: malate synthase (At5g03860). The developmental stages are germinated seed, seedling, young rosette, botting, young flower, developed flower, flowers and siliques, mature siliques, and senescence, respectively.

We obtained the data to support our identification of the Arabidopsis isoforms involved in the glyoxylate cycle in Arabidopsis, soybean and maize from Genevestigator (https://genevestigator.com), which is also a useful analysis tool and a database of transcriptomic data. The relatively small number of samples/experiments for soybean and maize in the database meant that we could not define a clear trend of aconitase gene expression during plant development. However, for Arabidopsis, we were able to identify the trend of the expression of the three aconitase genes in parallel with the gene expression of isocitrate lyase and malate synthase, as their products are the marker enzymes of the glyoxylate cycle/glyoxysomes. The aconitase gene that expresses similarly to isocitrate lyase (ICL) and malate synthase (MLS) genes is *ACO3*, although the three aconitase genes do not show relevant differences in expression. The heatmap of the gene expression in the nine developmental stages is shown in Figure 3A, while a scatterplot (log2 values) of the five gene expressions is given in Figure 3B. The relatively high expression of all three aconitase genes supports the importance of citrate metabolism in plant cells [54].

Further experiments with young Arabidopsis seedlings including knockout mutants need to be carried out in the future, to clearly demonstrate where the three aconitase isoforms are localized and which isoform(s) belong to the glyoxylate cycle. However, Arabidopsis seed/seedlings are somewhat “difficult” matrices to conduct experiments regarding cellular organelles fractionation on, due to the small size of the seeds and because the fats are distributed in various tissues and in cotyledons, endosperm, seed coat, radicle and hypocotyl [62]. The sunflower, which is an oil crop, could be a future plant model, because the genome sequencing work for *Helianthus annuus* is approaching completion [63] and because of the availability of extensive transcriptomic data (ePlant Sunflower) [64]. Sunflower oilseeds store lipids in their cotyledons, which during germination are tender enough to allow a homogenate suitable for sucrose gradient fractionation. In addition, the use of sunflower seeds means that abundant experimental material is always available.

## 4. Conclusions

The answer to the title’s question is not shrouded in Hamlet philosophical implications and can be summarized simply with a “not to be”. The data examined further demonstrate that the enzyme aconitase does not occur in glyoxysomes, although this has not been fully acknowledged in the literature. Finally, a paper published in May 2020 [65] revealed the presence of an Arabidopsis mitochondrial citrate/isocitrate/aconitate (AtSCF1) carrier exhibiting a higher transport affinity for tricarboxylates than dicarboxylates. A lower expression of this carrier reduces seed germination in antisense lines, suggesting that such a carrier could be involved in storage lipid mobilization, which catalyzes citrate/isocitrate or citrate/succinate exchanges. These data also indirectly support the role of a cytosolic aconitase in the plant glyoxylate cycle but suggest the possibility of very close coordination between glyoxysomes and mitochondria in the use of storage lipids during oilseed germination.

We revisited the issue of NAD reduction in glyoxysomes and discussed the role of the malate dehydrogenase in the glyoxylate cycle, re-iterating that within the organelle, malate dehydrogenase catalyzes the reduction of oxaloacetate to malate with the oxidation of NADH to NAD+, and a cytosolic malate dehydrogenase catalyzes the conversion of malate to oxaloacetate and the reduction of NAD+ to NADH. This means that the glyoxylate cycle is completed with two enzymes localized outside the organelle which contribute to produce malate in abundance within the glyoxysomes and oxaloacetate in the cytosol. We look forward to further evidence on this.

## Figures and Tables

**Table 1:** Prediction of the intracellular localization of Arabidopsis aconitase protein by different available databases.

Database/Program	ACO1 * (At4g35830)	ACO2 * (At4g26970)	ACO3 * (At2g05710)
The Plant Proteome Database (https://ppdb.tc.cornell.edu)	mitochondria; cytosol	mitochondria	mitochondria; cytosol
SUBcellular localisation database for Arabidopsis proteins—SUBA (https://suba.plantenergy.uwa.edu.au)	mitochondria; cytosol	mitochondria	mitochondria
Protein Localization Database (https://www.rostlab.org/services/locDB/index.php)	mitochondria; cytosol	mitochondria	mitochondria
Organelle DB (http://labs.mcdb.lsa.umich.edu/organelledb/index.php)	mitochondria	mitochondria	mitochondria

* The acronyms follow the nomenclature of Arnaud et al. [35].
